# Nutritional strategies of high level natural bodybuilders during competition preparation

**DOI:** 10.1186/s12970-018-0209-z

**Published:** 2018-01-15

**Authors:** A. J. Chappell, T. Simper, M. E. Barker

**Affiliations:** Food and Nutrition Group, Sheffield Business School, Sheffield Hallam University, Howard Street, Sheffield, S1 1WB UK

**Keywords:** Bodybuilders, Calories, Competition, Contest preparation, Dieting, Energy restriction, Natural, Nutrition, Supplementation, Physique

## Abstract

**Background:**

Competitive bodybuilders employ a combination of resistance training, cardiovascular exercise, calorie reduction, supplementation regimes and peaking strategies in order to lose fat mass and maintain fat free mass. Although recommendations exist for contest preparation, applied research is limited and data on the contest preparation regimes of bodybuilders are restricted to case studies or small cohorts. Moreover, the influence of different nutritional strategies on competitive outcome is unknown.

**Methods:**

Fifty-one competitors (35 male and 16 female) volunteered to take part in this project. The British Natural Bodybuilding Federation (BNBF) runs an annual national competition for high level bodybuilders; competitors must qualify by winning at a qualifying events or may be invited at the judge’s discretion. Competitors are subject to stringent drug testing and have to undergo a polygraph test. Study of this cohort provides an opportunity to examine the dietary practices of high level natural bodybuilders. We report the results of a cross-sectional study of bodybuilders competing at the BNBF finals. Volunteers completed a 34-item questionnaire assessing diet at three time points. At each time point participants recorded food intake over a 24-h period in grams and/or portions. Competitors were categorised according to contest placing. A “placed” competitor finished in the top 5, and a “Non-placed” (DNP) competitor finished outside the top 5. Nutrient analysis was performed using Nutritics software. Repeated measures ANOVA and effect sizes (Cohen’s *d*) were used to test if nutrient intake changed over time and if placing was associated with intake.

**Results:**

Mean preparation time for a competitor was 22 ± 9 weeks. Nutrient intake of bodybuilders reflected a high-protein, high-carbohydrate, low-fat diet. Total carbohydrate, protein and fat intakes decreased over time in both male and female cohorts (*P* < 0.05). Placed male competitors had a greater carbohydrate intake at the start of contest preparation (5.1 vs 3.7 g/kg BW) than DNP competitors (*d* = 1.02, 95% CI [0.22, 1.80]).

**Conclusions:**

Greater carbohydrate intake in the placed competitors could theoretically have contributed towards greater maintenance of muscle mass during competition preparation compared to DNP competitors. These findings require corroboration, but will likely be of interest to bodybuilders and coaches.

**Electronic supplementary material:**

The online version of this article (10.1186/s12970-018-0209-z) contains supplementary material, which is available to authorized users.

## Background

In competitive bodybuilding, athletes are judged on their muscularity (muscle size), conditioning (the absence of body fat) and symmetry (muscular proportion). In order to achieve the required physique, athletes undertake fat loss regimes, whilst attempting to maintain lean body mass (LBM) accrued prior to the fat loss period [1, 2]. Athletes and their coaches use a combination of resistance training, cardiovascular exercise, calorie restriction, supplementation and peaking strategies in order to obtain a competition-ready physique [3]. Bodybuilders preparing for competition usually follow self- or coach- prescribed diets, which often are comprised of a limited and repetitive food regime, with the sole aim of supplying specific amounts of protein, fat and carbohydrate [3–8]. Following these stringent dietary approaches is common practice and connects with the notion of being “hardcore” celebrated amongst bodybuilders [9]. Although broad recommendations exist for both nutrient intakes and exercise prescription [10–12], these recommendations are theoretical, imprecise, and open to interpretation. There is also a paucity of applied research on high level bodybuilders.

Recently a meta-analytic study combined 18 separate studies on the dietary intake of bodybuilders [13]. This study reported that male competitors consumed on average 3292 kcal per day during contest preparation, with 52% of that energy coming from carbohydrate, 28% from protein, and 22% from fat. Female competitors by way of comparison consumed 1739 kcal per day with 59% energy from carbohydrate, 28% energy from protein and 12% energy from fat. Although the meta-analysis incorporated 385 participants, the majority of the studies were published in the 1980s and 1990s and were non-specific about participants’ phase of training, which may be ‘off-season’ (prior to beginning contest preparation), during contest preparation (often a period of 8–24 weeks before competition), or 1 week from competition (the immediate pre-contest or peaking phase). The frequent use of androgenic anabolic steroids (AAS) amongst competitive bodybuilders also confounds identification of optimal nutritional strategies and training regimes. Indeed one third of the studies included in the meta-analysis reported AAS use by athletes [13]. Furthermore, the practices employed by athletes in the new physique categories, such as men’s physique, figure/ athletic, sports/fitness and swimsuit/bikini, which emphasize beauty rather than muscularity have not been scrutinised. Moreover, the lack of scrutiny of the practices employed within the aforementioned divisions may mislead bodybuilders as to what are the most effective strategies for competitive bodybuilding.

Within the United Kingdom (UK), the British Natural Bodybuilding Federation (BNBF) runs nine regional qualifying competitions; the regional qualifiers culminate in a UK final championship, where the overall winner is awarded professional status. This cohort provides an excellent opportunity to study the nutritional practices of a high level group of natural bodybuilders. The strategies employed by the most successful natural bodybuilders can be compared to recommendations [11], which include protein intake of between 2.3 and 3.1 g/kg of LBM, fat intake of 15 to 30% of total calories, with the remaining calories from carbohydrate and a weekly weight loss of 0.5 to 1% of bodyweight (BW) [11]. Here we report the results of a recent cross-sectional study investigating the nutritional strategies of natural bodybuilding competitors at the BNBF finals.

## Methods

### Design

Both male and female bodybuilders participating in the BNBF finals were included in the study. All competitors qualified for the UK final competition by winning their respective weight or age class at a regional qualifying event or were invited at the judge’s discretion; providing they had also been placed in the top three of their weight/ age category. All qualifying class winners were subject to drug testing based on urine analysis; targeted drug testing of other non-placed athletes was also carried out. Furthermore, all class winners at the final BNBF final were subject to the same drug testing criteria, and all competitors signed a waiver declaring their compliance with the World Anti-Doping Agency Code [14, 15]. A certified WADA laboratory (The Sports Medicine Research and Testing Laboratory, Salt Lake City, USA) carried out all drug testing. A qualified polygrapher polygraphed all competitors prior to taking part in the competition as an additional method to verify natural status.

The study was advertised via the BNBF social media page, and registered competitors were recruited in person by the first author at the outset of the UK finals. All potential participants were fully informed of the study aims and methods via a participant information sheet; those agreeing to participate provided written informed consent. Participants then completed a 34-item questionnaire (see Additional file 1). The questionnaire inquired about dietary and training habits, and body weight change at three time points throughout contest preparation (start, middle and end). Participants retrospectively recorded their typical food intake over a 24-h period in grams and/or portions. Missing questionnaire data and clarification about foods consumed/portions were followed up via email. The questionnaire also included items relating to the regular use of a coach, and “Cheat Meal” consumption. A “Cheat Meal”, is when competitors veer from their self- or coach-prescribed diet. Refeeds are strategies where competitors consume a known amount of energy in addition to their prescribed dietary intake, in the belief that it increases metabolic rate based on information from popular magazines and websites [16].

Results are reported separately for the male and female cohort as well as for participants who placed in the top 5 (placed) and those who were placed out of the top 5 of their class (Did Not Place (DNP)). All male competitors were from the bodybuilding category, while the female competitors were recruited from the bodybuilding, athletic and figure classes. Both the athletic and figure class emphasises less muscularity than female bodybuilding, with bodyfat levels distinguishing the two categories: lower (athletic) or higher (figure) bodyfat.

### Participant characteristics

Competitors reported their offseason (prior to starting their contest preparation) and competition (the day prior to taking part in the competition) bodyweights. Total weight loss and percentage weight loss were calculated as the difference between the start and end body weight. Body Mass Index (BMI) (kg/m^2^) was calculated from self-reported height and end body weight, body fat percentage (BF%) and method used to estimate was based on self-reported accounts. Only competitors who reported a BF% measured using callipers (*n* = 9) were included in the calculation of mean BF% and fat free mass index (FFMI) [17]. The FFMI was calculated based on the estimated fat free mass (FFM) at the end point of the contest preparation and expressed as kg/m^2,^.

### Dietary analysis

Nutritional analysis of contest diets was performed using the Nutritics Nutrition Analysis Software (version 4.267 Academic Edition, Nutritics, Dublin, Ireland). Macronutrient intake in grams per kg of bodyweight per day (g/kg BW) and energy intake in kilocalories per kg of bodyweight (kcal/kg BW) was calculated for the start and end of the diet period, based on competitors’ self-reported bodyweight. Macronutrients from dietary supplements were included in the analysis based on manufacturer’s specifications from brand websites. The mean number of food items consumed by a competitor at each phase of preparation was counted. The percentage of the diet made up of specific food groups was based on the European Food Safety Agency food classification system for dietary reporting [18]. Any food group making up less than 1% of the dietary intake was placed in the other ingredients category. Beverages, including water, teas and coffees were excluded from the food group analysis. Consumption of sugary soft drinks was not reported by any competitor and so do not feature in this analysis.

### Supplements

Supplements were split into 12 different categories reflecting those most commonly utilised by competitors: “Multivitamin”, “Vitamin C”, “Vitamin D”, “Mineral or Joint Supplement”, “Omega 3”, “Pre-Workout”, “Protein Powder”, “Branch Chain Amino Acids (BCAA)”, “Creatine supplement (either directly or part of another supplement)”, “Individual Amino”, “Fat Burners” and “Miscellaneous” (supplements used too infrequently to be awarded their own category).

### Statistical analysis

Data analysis was performed using the statistical analysis package IBM SPSS (version 24). Successful bodybuilders (placed) and unsuccessful bodybuilders (DNP) were compared for dietary intake (total energy intake (kcal per day), and total nutrient intake (g per day), using a repeated measures analysis of variance (ANOVA). Energy and nutrient intake adjusted for bodyweight (kcal/kg BW; g/kg BW) was log-transformed to account for skewed data and was then analysed by repeated measures ANOVA. The effect of time, contest place and time ⨯ contest place was examined. Mauchly’s test of sphericity was applied to data to examine if sphericity was violated and if this was the case the Greenhouse-Geisser estimate was utilised. For ease of interpretation we report the data as energy and nutrient intake adjusted for bodyweight. Hypothesis testing for categorical variables was performed using a Pearson Chi-Square for: contest outcome (placed and DNP), use of coaching and consumption of “Cheat Meals”. Independent T-Tests were used to identify if contest outcome (placed and DNP) was related to: i) years training, ii) years competing, iii) starting weight, iv) end weight, v) weight loss, vi) % weight loss, vii) weeks dieting, viii) weight loss per week, ix) caffeine intake, x) number of meals, and xi) fluid intake. Statistical significance was declared where *P* < 0.05 and the null hypothesis was rejected. Cohen’s *d* practical significance was calculated for the effect of contest outcome (placed and DNP) on energy and macronutrient intakes for male bodybuilders (as opposed to the multiple female competitive classes) for g/kg BW, and kcal/kg BW. Pooled standard deviations were used to calculate Cohen’s *d* and effect sizes were multiplied by an adjustment factor 0.975, to correct for bias to produce *d*. Effect size cut-offs were defined as 0.2, 0.5, and 0.8 for small, medium and large effect sizes respectively.

## Results

### Participant characteristics

Fifty-one bodybuilders (35 male, 16 female) participating in the BNBF finals volunteered for the study, comprising just over a third of the competition entrants (*n* = 143). All male competitors were from the bodybuilding category, while female competitors were from 3 separate categories: bodybuilding (*n* = 3), figure (*n* = 9) and athletic (*n* = 4). The female categories were pooled for analysis. One male competitor was excluded due to failing the pre-competition polygraph. The cohort included 6 competitors who had competed in a world amateur championship (4 of whom were former world amateur champions). The cohort also included 4 competitors who had previously placed at a BNBF UK final, 3 competitors who had won a regional overall BNBF title and 14 who had previously won a class at a BNBF regional qualifier. Complete dietary and training information was available for 32 male and 15 female competitors.

The majority of the competitors (66.7% men, and 88.0% women) used a coach for guidance with training, nutrition, posing and feedback on their physique. Male competitors who were placed did not differ from those DNP in use of a coach (60.0% versus 59.1% respectively) (χ^2^ (1) 0.550, *P* = 0.46). There was also no difference (χ^2^ (1) 0.163, *P* = 0.69) in the employment of a coach amongst female competitors who placed (100.0%) and DNP (78.0%). Participant characteristics, including age, years training and competing, bodyweight at the start of contest preparation and length of time following a diet are presented in Table 1.

**Table 1:** Characteristics of competitive bodybuilders recruited from the British Natural Bodybuilding Federation’s British championship

	Male competitors					Female competitors					Male		Female	
	Placed	SD	DNP	SD	P result	Placed	SD	DNP	SD	P result	Mean	SD	Mean	SD
Age	36.1	17.4	31.8	10.1	0.40	33.7	9.1	34.7	11.5	0.86	32.2	14.0	34.2	10.2
Years training	14.2	14.1	10.9	7.9	0.40	8.1	10.3	4.0	2.7	0.28	11.6	11.1	6.0	7.3
Years competing	3.5	2.6	2.7	2.2	0.34	1.9	0.7	2.3	1.3	0.40	3.1	2.4	2.1	1.1
Competitions this season	2.3	0.8	3.1	1.9	0.18	2.3	1.0	2.9	1.5	0.38	2.6	1.5	2.6	1.2
Diet start weight (kg)	82.5	10.4	89.2	10.4	0.08	64.7	9.5	62.9	4.9	0.63	85.4	10.8	63.8	7.1
Diet end weight (kg)	73.1	8.5	77.9	8.7	0.12	54.5	7.0	54.8	3.9	0.91	75.1	8.8	54.7	5.3
Total weight loss (kg)	9.4	5.6	11.3	5.2	0.32	10.2	4.3	8.1	3.4	0.29	10.3	5.4	9.1	3.9
Weight loss per week (kg)	0.6	0.7	0.5	0.7	0.74	0.4	0.1	0.5	0.4	0.81	0.5	0.7	0.5	0.3
% Weight loss	11.1	5.8	12.5	5.2	0.48	15.4	5.2	12.7	4.9	0.30	11.8	5.5	14.0	5.1
End BMI (kg/m^2^)	24.0	1.8	24.6	1.9	0.40	20.4	1.6	20.6	1.4	0.79	24.3	1.8	20.5	1.4
Diet length (weeks)	23.9	11.3	23.2	8.2	0.17	24.1	8.4	22.4	9.2	0.71	24.9	9.6	23.3	8.6

P result, difference in means between Place and DNP. Male competitors, Placed n - 15, DNP n - 18, Male Mean n - 32, Female Competitors, Placed n - 7, DNP n - 9, Female Mean n - 16. Data analysed using a student t test where *P* < 0.05 equals statistical significance

*Abbreviations*: *SD* standard deviation, *Placed* finished in the top 5 of the competition, *DNP* did not place in the top 5 of the competition, *BMI* body mass index

### Body composition estimation

Ten male and 5 female competitors provided a self-reported BF% along with the method they used to estimate/calculate it. Skin callipers were the most popular method of assessment with 53% of competitors (5 male and 4 female) using this method. Thirteen percent of competitors estimated BF% by looking in the mirror, while the method employed by the remaining 34% was not stated. Competitors did not specifically report whether a trained professional performed the skin calliper tests. An estimate of FFMI was calculated for male and female competitors who reported BF% measured by callipers. The estimated FFMI for placed males was 22.74 ± 2.55 kg/m^2^, with two competitors exceeding 25.0 kg/m^2^. In female competitors, the mean estimated FFMI was 18.1 ± 1.95 kg/m^2^.

### Dietary intake

#### Macronutrients, and diet diversity

The mean macronutrient and energy intakes reported as g/kg BW and kcal/kg BW at the start and end of contest preparation are presented in Table 2. Macronutrient and energy intakes presented as medians and interquartile ranges, are provided as an additional file (see Additional file 2). Analysis of log-transformed energy and nutrient intake adjusted for bodyweight indicated no significant difference in protein, carbohydrate, fat and energy intakes between placed and DNP competitors. Amongst both male and female competitors there was a significant decrease in energy intake over time (Male *P* < 0.001, Female *P* = 0.01), while there was also a non-significant decrease in carbohydrate intake over time amongst male competitors (*P* = 0.054). Results of the Cohen’s *d* adjusted effect size testing for macronutrient and energy intake scaled to bodyweight indicated a small to medium effect on competitive outcome for g/kg BW protein, in male competitors who placed compared to DNP (*d* = start 0.49, 95% CI [− 0.25, 1.30], end 0.60, 95% CI [− 0.15, 1.37]). A similar outcome was noted for carbohydrate intake between males who placed and DNP (*d* = start 1.02, 95% CI [0.22, 1.80], end 0.35, [− 0.40, 1.10]). Small effect sizes were noted for both fat as g/kg BW (*d* = start 0.24, 95% CI [− 0.97, 0.52], end 0.25, 95% CI [− 0.50, 1.0]) and energy as kcal/kg BW (*d* = start 0.71, 95% CI [− 0.04, 1.47], end 0.65, 95% CI [− 0.09, 1.41]) between males who placed and DNP.

**Table 2:** Macronutrient (g/kg bw) and energy intake (kcal/kg bw) of competitive bodybuilders during contest preparation: mean daily intake with standard deviations

	Male							Female					
	Diet phase	Placed	SD	DNP	SD	Mean	SD	Placed	SD	DNP	SD	Mean	SD
Protein	Start	3.0	1.0	2.7	0.6	2.8	0.8	2.7	0.6	2.7	0.5	2.7	0.5
	End	3.3	0.9	2.7	0.8	3.0	0.8	2.8	1.0	2.9	0.6	2.8	0.8
Carbohydrate	Start	5.1	1.9	3.7	0.9	4.4	1.4	3.7	2.2	4.0	0.9	3.9	1.6
	End	4.6	2.2	3.6	2.7	4.1	2.4	3.5	1.6	3.0	1.5	3.3	1.5
Fat	Start	0.8	0.3	0.9	0.6	0.8	0.4	0.7	0.3	0.9	0.4	0.8	0.4
	End	0.6	0.3	0.6	0.3	0.8	0.8	0.5	0.1	0.7	0.4	0.6	0.3
Energy	Start	38.2	11.1	32.6	7.5	35.4	4.5	30.9	7.4	33.8	6.3	32.5	6.7
	End	35.8	8.6	29.7	10.1	32.7	5.5	29.3	3.9	29.7	7.6	29.5	10.8

*Abbreviations*: *SD* standard deviation, *Placed* achieved top 5 of the competition, *DNP* did not place in top 5 of the competition

Mean macronutrient and energy intakes reported as total g per day and kcal per day for the start, middle and end of contest preparation are presented in Table 3. Total carbohydrate, fat and energy intakes significantly declined over time (*P* < 0.05) in both the female and male groups, while there was a non-significant decrease in protein intake (*P* = 0.09) amongst male competitors. The mean number of food items consumed by male competitors was: start 11.5 ± 3.6, middle 9.7 ± 4.7, and end 10.0 ± 3.5. Amongst female competitors the mean number of food items consumed was: start 12.3 ± 3.4, middle 13.2 ± 4.0, and end 10.6 ± 3.9. The contribution of different food groups to the competitors’ diets is presented in Table 4. No competitor reported consuming composite diet dishes, food imitates (meat and dairy alternatives), sugar sweetened beverages or alcohol at any time point during their preparation.

**Table 3:** Total macronutrient intake of competitive bodybuilders during contest preparation: mean daily intake with standard deviations

	Diet phase	Placed	SD	DNP	SD	Mean	SD	*P* value time	*P* value result	*P* value time x result
Male										
Protein (total g per day)	Start	254.0	92.7	232.3	52.3	242.4	73.4			
	Middle	252.9	76.4	231.2	52.2	240.9	63.9	0.09	0.20	0.73
	End	243.7	64.2	205.2	54.4	222.4	61.1			
Carbohydrate (total g per day)	Start	431.1	165.1	323.6	78.9	373.8	135.7			
	Middle	392.8	142.5	288.0	106.5	335.0	132.6	0.01	0.14	0.51
	End	340.6	156.5	268.6	185.0	300.8	173.7			
Fat (total g per day)	Start	64.7	26.0	75.8	48.7	70.6	39.5			
	Middle	61.7	21.7	69.4	48.6	65.9	38.5	0.01	0.57	0.57
	End	45.4	20.3	44.8	23.9	45.1	22.0			
Energy (total kcal per day)	Start	3214.8	1023.4	2824.6	587.5	3006.7	829.0			
	Middle	3039.5	796.6	2629.9	575.6	2813.5	701.7	0.01	0.20	0.83
	End	2660.6	571.3	2231.0	659.9	2423.6	648.4			
Female										
Protein (total g per day)	Start	172.0	28.3	177.1	22.5	174.7	24.6			
	Middle	168.3	34.1	166.3	24.7	167.2	28.4	0.93	0.63	0.56
	End	150.9	44.1	166.8	29.3	159.3	36.5			
Carbohydrate (total g per day)	Start	243.7	155.5	274.0	66.0	259.9	113.1			
	Middle	220.9	126.3	226.0	72.1	223.6	97.1	0.01	0.90	0.54
	End	196.7	99.2	179.0	84.0	187.3	88.5			
Fat (total g per day)	Start	44.3	18.8	59.2	28.0	52.3	24.5			
	Middle	44.1	19.2	52.7	19.7	48.7	19.3	0.02	0.17	0.64
	End	28.6	6.1	44.0	22.0	36.8	17.9			
Energy (total kcal per day)	Start	2000.8	551.7	2269.0	423.5	2143.8	489.2			
	Middle	1898.3	479.8	1986.7	405.1	1945.4	405.1	0.01	0.76	0.70
	End	1598.9	256.8	1734.4	363.0	1671.1	363.0			

P time, difference in means over time (start, middle, end), P result, difference in means between Place and DNP. Time x Result interaction between diet over time and result. Differences in macronutrients and energy measured by repeated measures ANOVA. Statistical significance assumed where *P* < 0.05

*Abbreviations*: *SD* standard deviation, *Placed* achieved top 5 of the competition, *DNP* did not place in top 5 of the competition

**Table 4:** Percentage food group intake of competitive bodybuilders during contest preparation

		Cereals and cereal based products					Nuts, oilseeds and legumes		Meat and meat products						Animal and veg fats and oils			
	Diet Phase	Cereals	Bread	Dairy	Veg	Tubers	Nuts and Seeds	Legumes	White	Red	Processed	Fruit	Eggs	Marine	Veg	Animal	Sugar	Other
Male	Start	17.7	0.4	15.2	11.3	9.5	10.0	0.0	9.1	3.9	1.3	7.4	5.2	4.3	2.6	0.9	0.0	0.9
	Middle	17.6	0.0	11.9	11.4	11.4	9.3	0.0	9.3	3.6	0.0	7.3	5.7	8.3	2.1	0.5	0.0	1.0
	End	15.3	0.5	14.7	15.3	10.5	6.8	0.0	9.5	2.1	0.0	8.4	6.3	7.4	2.1	0.5	0.0	0.0
Female	Start	22.0	2.5	13.6	16.1	10.2	6.8	0.0	8.5	3.4	0.0	5.9	3.4	5.9	0.9	0.0	0.9	0.0
	Middle	18.9	1.9	11.3	15.1	8.5	9.4	0.0	5.7	2.8	0.0	9.4	6.6	8.5	0.9	0.0	0.9	0.0
	End	19.8	1.9	13.2	13.2	12.3	6.6	0.0	9.4	0.9	0.0	5.7	6.6	8.5	0.9	0.0	0.9	0.0

Dairy products includes whey and a casein supplements, Processed meats include sausages, bacon, pies meat pastries etc., Fruit includes fruit products, Eggs includes egg products including egg protein isolate, Marine, includes fish, seafood, amphibians reptiles and invertebrates. Sugar includes confectionary and water- based sweet desserts

*Abbreviations*: *Veg* vegetable

Competitors frequently used supplements over the course of contest preparation; these data are presented in Table 5. The consumption of caffeine, fluids and number of meals a competitor consumed were compared between placed and DNP competitors. There was no statistically significant difference (t (25) = 0.51, *P* = 0.62) in daily caffeine consumption between males who placed (360 ± 198 mg) and DNP (283 ± 153 mg). There was also no difference (t (13) = 0.80, *P* = 0.44) in daily caffeine consumption between females who placed (277 ± 158 mg) and DNP (232 ± 173 mg). Mean daily caffeine consumption was 322 ± 176 (range 0 to 1384 mg) and 252 ± 161 (range 0 to 618 mg) mg per day for males and females respectively. Fluid intake for males who placed and DNP place was 4.5 ± 1.7 and 4.5 ± 1.6 l per day, with no difference between the groups (t (31) = 0.90, *P* = 0.37). In females the fluid consumption was 4.4 ± 2.3 and 3.8 ± 1.1 l per day, with no significant difference between the two groups (t (14) = 0.61, *P* = 0.55). Mean consumption of fluids for males and females was 4.5 ± 1.6 and 4.1 ± 1.7 l per day respectively.

**Table 5:** Supplement usage of competitive bodybuilders during contest preparation: mean daily intake with standard deviations

	Male	SD	Female	SD
Mean number of supplements used	7.0	0.8	5.4	3.2
Protein powder (%)	75.0	11.8	88.9	15.7
Multivitamin (%)	53.3	28.3	60.0	14.1
BCAA supplement (%)	49.4	14.9	53.5	12.8
Creatine supplement (%)	48.3	2.4	50.8	9.0
Fat burners (%)	47.8	11.0	36.5	11.2
Individual amino (%)	42.2	3.1	31.0	3.3
Pre-workout (%)	42.2	3.1	19.9	12.4
Omega 3 (%)	39.4	0.8	46.6	12.8
Carb supplement (%)	36.7	4.7	0.0	0.0
Mineral or joint supplement (%)	27.2	0.8	31.0	3.3
Vitamin C (%)	23.9	5.5	28.5	22.6
Vitamin D (%)	21.1	1.6	11.1	15.7
Miscellaneous supplement (%)	42.8	5.5	5.6	7.8

Values expressed as a percentage of the population who utilised a particular supplement. Miscellaneous supplement, supplements that were used too infrequently to be designated as a category

*Abbreviations*: *SD* standard deviation, *BCAA* branch chain amino acids

The number of daily meals consumed by competitors was counted, there was a trend (χ^2^ (5) 2.40, *P* = 0.10) for males who placed to consume more meals per day than those who DNP, 6.5 ± 0.9 vs 5.9 ± 1.1 respectively. There was no significant difference (χ^2^ (2) 0.93, *P* = 0.23) in the number of meals consumed per day between females who placed and DNP, (6.1 ± 0.7 vs 6.7 ± 0.8). The mean number of meals consumed per day was 6.2 ± 1.0 and 6.4 ± 0.8 for male and females respectively. Of the 32 male competitors, 10 (31.2%) reported the consumption of “Cheat Meals”. “Cheat Meal” consumption was less common amongst placed competitors with only 3 (20.0%) stating they used “Cheat Meals”, while 7 (38.0%) of DNP competitors reported consuming a “Cheat Meal”. A single placed competitor (5.0%) reported utilising a refeed strategy. There was no difference in “Cheat Meal” consumption between males who placed and DNP (χ^2^ (2) 2.82, *P* = 0.28). Of the 16 female competitors, 8 (50.0%) consumed a “Cheat Meal” during contest preparation. Reports of “Cheat Meal” consumption were less frequent amongst competitors who placed, 1 (6.3%) compared to those who DNP, 3 (18.8%), while 1 placed (6.3%) competitor and 3 DNP (18.8%) competitors specified employing refeed strategies. However there was no significant difference between the groups (χ^2^ (2) 2.62, *P* = 0.19).

## Discussion

This study is novel in providing insight on the nutritional strategies of high-level competitive natural bodybuilders. Although other studies have claimed to recruit high-level or elite natural bodybuilders, their definition of elite has been less stringent than the present investigation [3, 5]. We found no significant difference in dietary intake between the placed and DNP competitors. In spite of this null effect, results of practical significance testing suggest carbohydrate consumption in the early stages of contest preparation may influence competitive outcome in the male bodybuilders. We also report that high level natural bodybuilders consume more energy, particularly from carbohydrate than previous accounts of natural bodybuilders [3–8]. As bodybuilders approached competition, energy intake is reduced primarily through a reduction in carbohydrate and fat intake, with protein intake remaining constant throughout contest preparation. Accounts of body composition measurement show that bodybuilders often employ subjective methods to estimate BF% [17]. Finally, we report on supplement and caffeine intake and note high consumption compared to publicly prescribed safety recommendations for caffeine.

### Body composition and the fat free mass index

Skin callipers were the most commonly used methods for estimating BF% amongst competitors, while subjective methods based on appearance were also reported. Objective measurements of BF% from competitive bodybuilders have been reported previously; these data suggest ranges from 4.1 to 10.9% and 8.6 to 11.3% in males and females respectively [2, 6–8, 19–21]. Interestingly in a sport concerned with achieving a low body fat, the majority of competitors did not report BF%, with a proportion using visual methods to estimate BF%. It is possible that the lack of reporting of BF% may reflect a greater emphasis placed on the appearance of low body fat in bodybuilding rather than objectively obtained measures.

A FFMI above 25 kg/m^2^ is suggested as an upper limit for muscle accretion, without the use of AAS [16]. This threshold, however, is based on photographic estimates of pre 1959 Mr. America bodybuilders and the objective measurement of 157 gymnasium users so should be interpreted with some caution [17]. We provided an estimate of the mean FFMI based on competitors who reported BF% using callipers of 22.7 kg/m^2^, this is higher than the 21.8 kg/m^2^ reported in the study of gymnasium users [16]. Interestingly, two male competitors in this study had a FFMI above the 25 kg/m^2^, (25.73 and 25.15 kg/m^2^) natural threshold based on the pre 1959 Mr. America winners. We did not measure FFM or BF% directly and we acknowledge the limitations of self-reported accounts of BF%; however, it is not inconceivable that there are high-level natural bodybuilders who exceed this theoretical FFMI 25.0 kg/m^2^ threshold. The mean FFMI of 18.1 kg/m^2^ reported in females was in agreement with previous estimates of 18.3 kg/m^2^ [21]. but greater than that of a recent case report [6]. No FFMI upper threshold has been proposed for females; however, a FFMI between 19.0 to 20.0 kg/m^2^ seems a reasonable objective starting point based on estimates of female populations [22].

### Dietary intake

#### Energy intake

As expected energy intake of male and female competitors was higher at the start of contest preparation compared to the end. Similar findings have been reported in previous observations [13, 23, 24]. Competitors reported reducing energy intake in stages over the course of their preparation with smaller differences from the start to the middle and middle to end of the diet. Similar strategies involving modest reductions in carbohydrate and fat consumption to facilitate weight loss has been reported elsewhere [4]. In contrast, two case studies of bodybuilders [5, 7] reported a reduction in energy intake between 882 to 1300 kcal/d from the start to the end of the competition preparation, compared to smaller reductions (554 kcal/d) in our placed males. Smaller reductions are intended to counteract metabolic adaptations to dieting, changes in energy requirements and preservation of LBM [11]. Both male and female competitors reported a high meal frequency. This may reflect the practical aspects of consuming large volumes of food combined with and belief that multiple meals may preserve more LBM, while contributing to greater appetite control [25–27].

Research indicates greater LBM preservation and exercise performance with slower versus faster weight loss, a 0.5 to 1% of BW per week is recommended for natural bodybuilding [10, 28]. Our cohort reporting dieting for on average 24.9 and 23.3 weeks in males and females respectively, compared to 14 weeks in the study by Robinson et al. [5], and 26 weeks in both Rossow et al. [3] and Kister et al. [4] studies. The weekly weight loss in the present study was estimated to be 0.46% per week in the placed males, compared to 0.5 [3], 0.7 [4] and 1.0% [5] in case studies. Fascinatingly, Petrizzo et al. [7] recently advocated weight loss of 0.5% BW per week for LBM preservation during natural bodybuilding contest preparation. In the present investigation, weight loss was 0.46% per week reflecting this recommendation; it seems likely that slower weight lost in our placed males may have resulted in greater preservation of LBM than the DNP males.

### Macronutrients

#### Carbohydrate

Carbohydrate was the most abundant macronutrient consumed across all the phases of the diet, in both male and female competitors. The majority of carbohydrates came from cereals, tubers, fruit, and vegetables. Confectionary items, such as sweets and water-based desserts, legumes and bread were consumed sparingly during contest preparations in agreement with previous accounts of bodybuilding menus [5, 7, 29]. Carbohydrate intake was reduced from the start to the end of contest preparation reflecting the practice seen in bodybuilding case studies [3–5]. Carbohydrate intake amongst placed males (5.1 g/kg BW) was similar to a meta-analysis of contest preparation bodybuilders (4.9 g/kg BW) [13]. However, intake was higher amongst placed male competitors in the weeks preceding the competition (end of the diet), 4.6 g/kg BW compared to previous reports (3.8 g/kg BW) [13]. Intake was also higher for male competitors compared to three recent case studies, where mean intakes were between 2.5 to 3.0 g/kg bw over 26 and 28 weeks [3, 4], and 1.2 to 1.4 g/kg bw over 14 weeks [5].

Carbohydrate energy in the female cohort (placed and DNP Start of diet: 3.7 and 4.0 g/kg BW), was higher than two case studies (3.4 g/kg BW [6] and 1.5 to 1.9 g/kg BW [7]) and a meta-analysis conducted on studies reporting intake amongst female bodybuilders over 30 years ago (3.1 g/ kg BW) [13]. Higher intakes have been reported in a recent case report of a dieting female physique competitor where intake was initially 5.0 g/kg BW [8] before decreasing to 1.8 g/kg BW by the end of the 6 month study period. Intakes of 5.0 g/kg BW have been reported in the final week of contest preparation, [13] which may reflect carbohydrate loading strategies in the final week of competition.

Carbohydrate intake amongst the placed males (4.6 to 5.1 g/kg BW) was in line with previous recommendations of 4–7 g/kg BW for bodybuilders aiming to maintain weight [30]. Cohens *d* effect size testing indicated carbohydrate intake at the start of the diet may have some impact on competitive outcome. Placed males consumed 1.0 to 1.4 g/kg BW more carbohydrate than those who DNP during this period. This equates to an additional 75 to 97.5 g carbohydrate (281.2 to 365.6 kcal) per day in a 75 kg male. This additional carbohydrate is a non-trivial amount as even modest resistance training protocols can deplete muscle glycogen concentrations between 24 to 82% [31, 32]. Furthermore, as little as 15 g of carbohydrate consumed during resistance training may increase performance in hypertrophy rep ranges [33]. Finally, the effect of isometric contractions on bodybuilders’ muscle glycogen concentrations should be considered. Bodybuilders routinely hold isometric contractions for between 6 to 60s for 10 to 30 min In preparation for competition [8]. By way of comparison 10 min of isometric exercise at 20% of maximum voluntary contraction for 10 s, with 10 s rest intervals can severely deplete muscle glycogen in type 1 fibres [34]. An adequate carbohydrate intake during contest preparation to maintain muscle glycogen should therefore be an important consideration for the natural bodybuilder.

Low carbohydrate diets are effective for weight loss, however they may result in a disproportionate loss of LBM [35–38]. An example of this can be seen in the study by Robinson et al. [5] where the athletes lost 43% of their LBM following a low carbohydrate diet. While higher carbohydrate diets utilised by other bodybuilding case studies resulted in smaller LBM loss, 21% and 32% [3, 4]. Multiple factors could have contribute to the LBM loss seen in Robinson et al. [5], however recent critiques have suggested more prudent dietary strategies should be employed for natural bodybuilding [7, 39]. Interestingly, the two female athletes involved in the Petzzaro et al. [7] and Rohrig et al. [8] case reports increased LBM and reduced fat mass in despite following a similar dietary approach to Robinson et al. [5]. With the exception of a case report which tracked the body composition of 6 physique athletes (4 male, 2 female) using AAS [40] increases in LBM and a reductions in fat mass amongst bodybuilders during contest preparations have been previously unreported [1, 3–6, 21, 41–43]. In spite of this finding evidently, there may be a threshold for carbohydrate intake, after which there is an increase in the rate of LBM loss regardless of protein intake or resistance training.

#### Protein

Protein constituted between 32.0 to 40.0% of total energy in both male and female competitor’s diets in the present study in line with previously reported data [12]. The main protein sources were from dairy, white meat, nuts and seafood in agreement with data from previous bodybuilding studies [5, 6, 29]. The high intake of protein from dairy reflects the prominence of protein powder in competitors’ diets. Red meat and eggs were consumed to a lesser extent than the aforementioned foods options. Protein intakes were between 2.7 g/kg BW and 3.3 g/kg BW amongst male and female competitors, similar to reports from case studies 2.2 to 3.5 g/kg BW [3–8].

The prioritisation of protein over other macronutrients during energy restriction is common practice amongst bodybuilders [3–8, 40]. High protein diets are known to spare LBM during energy deficits [43–46], maintain nitrogen balance and stimulate muscle protein synthesis (MPS) [47]. Protein is also satiating, which may improve dietary adherence during energy restriction [45]. Protein digestion is also known to have the greatest thermic effect of the three macronutrients. Approximately 20 to 30% of net energy is lost through dietary thermogenesis of protein (compared to around 3% for fat); this extra thermic effect may contribute to additional weight loss [36, 45].

Protein intake recommendations for strength-trained athletes during energy restriction are 2.0 g/kg BW, with 0.25 to 0.3 g/kg BW during the early recovery phase after exercise [48]. However, a recent systematic review has recommended higher levels of between 2.3 to 3.1 g/kg BW of LBM during severe calorie restriction [49], although this recommendation was based on only two empirical studies [36, 43]. Nevertheless it seems likely that protein intake amongst the competitors in this study was adequate for the preservation of LBM. This additional protein may be advantageous exploiting the thermic effect of food and satiation offered by additional protein. However, a recent experimental study found that additional protein energy was not realised in changes in body composition [37, 50].

#### Fat

Fat intake was the lowest amongst the three macronutrients, and like carbohydrate was reduced over time in favour of maintaining protein. There was a tendency for competitors in this cohort to favour low-fat diets. Food selection patterns emphasised this with oils and red and processed meats making up only a small percentage of the cohort’s overall diet. Although eggs were commonly consumed, the yolks were routinely discarded. Nuts and seeds, along with white meats, marine and cereals were the most prominent sources of fat in the athletes’ diet. Moreover, many athletes reported consuming an omega-3 fatty acid supplement, suggesting athletes favouring a diet higher in mono- and polyunsaturated fatty acids rather than saturates. Low fat food selection patterns have previously been reported [29].

The fat intakes recorded in the present study (start of diet: males 0.8 g/kg BW, females 0.8 g/kg BW) were similar to previous accounts reported by Spendlove et al. [13] for male (0.95 g/ kg BW) but higher than previous reports of female competitive bodybuilders (0.32 g / kg BW). Despite the seemingly low fat intake accounts as low as 8% of energy have been reported in the bodybuilding literature [51, 52]. Bodybuilder’s in the present study placed nutrients in a hierarchy, prioritising protein followed by carbohydrate for the aforementioned reasons. Low fat diets have however been cited to reduce testosterone concentrations during a calorie deficit [53, 54]. However, in a 11 week study of bodybuilders dieting for competition, plasma testosterone and IGF-1 concentrations decreased, despite subjects consuming a relatively high fat intake (energy 25% or (1.18 g / kg BW) [43]. Differentiating between the effects of fat and energy intake on hormone concentrations is clearly a challenge. The intakes recorded in this study in conjunction with high protein and high carbohydrate diets may therefore merit further investigation.

Finally, the low fat intakes seen here may reflect a paradigm shift between the present day, and the 1980’s and 90’s where the majority observational studies of bodybuilders were performed [13]. Likewise the results reported here may also reflect a difference in approach between British and American bodybuilders, with all six recent case studies opted for a higher fat approach [3–8].

### Supplementation and caffeine intake

Male and female competitors routinely consumed between 5 and 7 supplements during contest preparation. Protein powders, multivitamins, BCAA and creatine were the most commonly consumed supplements in agreement with previous observations of gymnasium users, athletes and bodybuilders [55–57]. Whey protein was routinely consumed at breakfast and post resistance training. In contrast, casein-based supplements were commonly consumed as the last meal of the day. The use of protein in this manner suggests nutrient timing strategies, the effectiveness of which has been called into question [58]. Branch chain amino acids are used as means of maximally stimulating MPS over the course of the day by providing a bolus dose of BCAA every few hours along with or between meals, for the preservation of LBM in what is commonly referred to as a ‘pulsing strategy’ [47]. However when the muscle full effect is considered, the use of BCAA as part of a high protein diet may offer little if any additional stimulation of MPS [59]. Moreover, the daily doses of BCAA (30 g) and beta-hydroxy-beta-methylbutyrate (2 g), consumed by the athletes in the Kistler et al. [4] case study failed to prevent a greater loss of LBM compared to Rossow et al. [3].

Specialist fat burning and pre-exercise supplements were also popular amongst this cohort. Pre-exercise supplements often contained combinations of vitamins, minerals, amino acids, creatine, caffeine and lactate buffers usually in the form of beta alanine, citrulline malate and arginine. The efficacy of these individual ingredients have been reviewed for natural bodybuilding [11]; however, their use in combination is largely unknown. In addition, because caffeine is a prominent ingredient in many of these supplements, competitors should consider the contribution these supplements make to their caffeine intake. This point is particularly pertinent when considering competitors may often consume large amounts of energy drinks and hot beverages [5–7]. Indeed, several competitors exceeded the 400 mg per day safety consumption limits specified by The European Food Safety Agency for caffeine. Although these consumption limits are based on a 70 kg individual, competitors should be aware that intakes above 9 mg /kg BW may be ergolytic as well as having unintended side effects [60, 61].

#### Limitations

There are limitations with the method used to assess dietary intake, under-reporting of habitual dietary energy intake is estimated to be between 18 to 54% in non-bodybuilding populations [62]. In particular carbohydrates tend to be under-reported, while protein intake over reported [62]. Likewise, foods that portray a negative health image such as confectionary are often under reported while foods that portray a positive health image such as fruits and vegetables are over reported [62]. The prevalence of under- or over reporting within bodybuilding population is unknown, however bodybuilders are known for their strict adherence to dietary plans [3–8]. The dietary recall only incorporated a single day’s intake at three arbitrary time points. As a result, competitors largely reported their dietary intake for training days, while intake on non-training days is likely to be lower. This bias likely resulted in inflated values for energy intake, furthermore strategies such as carbohydrate and calorie cycling were likely missed by the single day recall method. Despite the limitations of the method used to capture dietary intake we were able to detect a reduction of energy intake over time. Moreover, a single day’s intake may have led to misclassification of dietary diversity, although the diet diversity scores are agreement with previous accounts of bodybuilding menus.

The BF% values used to estimate the FFMI of competitors are based on self-reported accounts using skin callipers. Competitors did not report if these skinfold test were carried out by trained professionals. Although, the values reported were plausible (essentially they match with the values reported elsewhere amongst competitive bodybuilders taken form objective measures) for competitors competing in a national competition, we omitted to include them in the report along with the subjectively obtain values as they should not be regarded as accurate.

## Conclusions

The cross-sectional nature of this study makes it difficult to draw any definitive conclusion on what may be the best dietary approaches for contest preparation in natural bodybuilders. Our questionnaire was limited in scope and there are multiple variables that likely influence competitive outcome. Likewise, the sample size may have reduced our ability to detect statistical significance. In spite of these limitations, our study is distinctive in its applied nature attempting to correlate cross sectional data with competition outcome. The findings of this study required corroboration, and should be interpreting with caution. However, it is likely our findings will be of interest to pre-competition bodybuilders and those seeking to reduce fat mass while maintaining LBM who may wish to know strategies employed by bodybuilders in the placed cohort of a high level national bodybuilding competition.

This study also provides a contemporary account of current bodybuilding practices and provides additional evidence for the formation of research informed approaches to natural bodybuilding contest preparation. Future studies should focus on the use of standardizes body composition measurement techniques to assess changes in FFM during contest preparation. Furthermore, beyond case studies the literature is lacking in longitudinal studies of competitive bodybuilders. Larger scale longitudinal studies of competitive natural bodybuilders using with frequent sampling points would be of value to researchers in this field.

## Additional file

Additional file 1: Dietary assessment questionnaire used to carry out this research. (PDF 242 kb)
Additional file 2: Dietary intake of competitors adjusted for bodyweight expressed as Medians and Interquartile Ranges. (PDF 815 kb)
