# Supplementary material for: Nutritional strategies of high level natural bodybuilders during competition preparation
Source: J Int Soc Sports Nutr. 2018 Jan 15;15:4. doi: 10.1186/s12970-018-0209-z (PMC5769537; doi:10.1186/s12970-018-0209-z)
Supplement: Additional file 1: — Dietary assessment questionnaire used to carry out this research. (PDF 242 kb) [file 12970_2018_209_MOESM1_ESM.pdf]

Dietary Assessment of Natural Bodybuilding Population

Sheffield Hallam University

Food and Nutrition Group

BNBF
BRITISH NATURAL BODYBUILDING FEDERATION

Questionnaire Number:

Competitor Information

Please answer the following questions as accurately as possible. If you do not unsure about any of the questions please don't hesitate to ask for clarification.

Competitor Number:___________ Competitor Class:____________ Age:_________________

Number of year's training:____________ Number of year's competing:____________________

Competition weight:_________________ Weight at start of preparation:___________________

Height: _____ Bodyfat if known% (include method used to estimate):________________

____

Highest bodybuilding accolade (example: BNBF British Mens open lightweight 3rd 2013):

____

Do you have a background/training in health/fitness/medicine/ nutrition (if so specify):

____

Number of competitions this season (including this one):_______________________________

How many weeks did you diet for this competition (included qualifier if applicable):__________

If applicable, did you have a break after the qualifier, if so how long and what did you do?

____

Do you have someone to help you with your diet and training (please specify)?

____

PLEASE ANSWER THESE QUESTIONS RELATED TO YOUR USUAL CONTEST DIET

How would you describe your dietary approach? For example: High protein & low fat; High carbohydrate & low fat; High protein low carbohydrate; Ketogenic; Paleo; IIFYM Dirty, IIFYM Clean Gluten free, Zone, Atkins, Intermittent Fasting etc…

____

____

____

Did your dietary approach change during the preparation? For example did you move from a high carb to a low carb diet, or start carb cycling diet? Did you start with cheat meals and move away from cheat meals?

____

____

____

Did you use cheat meals, if so how often?: ____

What supplements did you use regularly rather than as one offs? for example:

Morning: Cod Liver oil (Seven Seas, High Strength), and multivitamin (Universal Animal Pack)

Pre-workout: fat burner (EQ nutrition, Nutrilean), BCAA, (USP labs Modern BCAA)

After training: Post workout shake (Extreme Nutrition, Build and Recover)

Pre-bed time: Micellar casein drink protein shake (CNP, Propeptide)

____

____

____

____

____

Fluids

How many Litres of water/drinks/cups of tea did you consume per day:____________________

____

Do you use artificial sweetened drinks? Does your consumption of these drink increase during the competition phase?

____

____

Did the amount you drink increased during your competition phase?______________________

____

Peaking

Athletes sometimes use peaking strategies in the weeks before contest. Is this something you do? If so please specify, for example: *"I usually use a carb and water loading plan. I eat five days' worth of my regular carbs over three days prior to the competition. I use mainly cakes and biscuits to do this. I also load on three times my regular water each day prior to contest, finally I consume 3,000mg of sodium every hour, 3 hours before I go on stage."*

____

____

____

____

____

____

Contest Day

Do you have a nutritional strategy for the contest day? For example: "*I like to follow my regular diet on contest day, however a few hours before I'll eat two packets of rice cakes and half a jar of diabetic jam before stage. I also like to drink a shot of brandy 20 minutes before stage*".

____

____

____

____

____

Post Competition

Have you made provisions for how you intend to approach your diet following the competition? For example: Reverse dieting, follow the previous contest dieting, three days of binging and then Clean eating etc.

____

____

____

Dietary Habits

Please detail the type and amount of food to the nearest gram you were consuming at the start (The first few weeks of your contest diet), halfway and final weeks of your contest prep (not including peak week).

Initial Diet	Middle Diet	End Diet
Example:<br>***M1:****100g of Weatabix in water, 60g of banana and 3 whole boiled eggs.40g of whey (Extreme Nutrition)*<br>***M2:*** *1 CNP pro flapjack (orange flavour), small apple 70g*<br>***M3:*** *150g of steamed cod,40g*	*Example:*<br>***M1:****50g of Weatabix in water, 60g of banana and 3 whole boiled eggs.40g of whey (Extreme Nutrition)*<br><br>***M2:*** *1 CNP pro flapjack (orange flavour), small apple 70g*	*Example:*<br>***M1:*** *60g of banana and 6 whole boiled eggs.50g of whey (Extreme Nutrition)*<br><br>***M2:*** *1 CNP pro flapjack (orange flavour), small apple 70g*

of spinach, 30g of green olives and 200g of boiled white potatoes<br>***M4:** Repeat meal 3*<br>***M5:** Repeat meal 1*<br>***Pre Workout:** 30g NO Explode Lime flavour (BSN)*<br>***Post Exercise:** 80g Build and Recover (Extreme Nutrition)*<br>***M7:**70g Low Fat Cottage Cheese (Tesco), 35g Pro Peptide (CNP)*<br>***Drinks:** 3 Cups of instant black coffee and sweeteners with meals (Kenco)*<br>*1 Can of Pepsi Max*	***M3:** 120g of steamed cod,40g of spinach, 30g of green olives and 150g of boiled white potatoes*<br>***M4:** Repeat meal 3*<br>***M5:** Repeat meal 1*<br>***Pre Workout:** 30g NO Explode Lime flavour (BSN)*<br>***Post Exercise:** 60g Build and Recover (Extreme Nutrition)*<br>***M7:**60g Low Fat Cottage Cheese (Tesco), 35g Pro Peptide (CNP)5g*<br>***Drinks:** 2 Cups of instant black coffee and sweeteners with meals (Kenco)*<br>*2 Can of Pepsi Max*	***M3:** 120g of steamed cod,40g of spinach, 30g of green olives and 100g of boiled white potatoes*<br>***M4:** Repeat meal 3*<br>***M5:** Repeat meal 1*<br>***Pre Workout:** 30g NO Explode Lime flavour (BSN)*<br>***Post Exercise:** 50g Build and Recover (Extreme Nutrition)*<br>***M7:**50g Low Fat Cottage Cheese (Tesco), 50g Pro Peptide (CNP)5g*<br>***Drinks:** 6 Cups of instant black coffee and sweeteners with meals (Kenco)*<br>*1 Can of Pepsi Max*

Cardiovascular Training

During your preparation the amount of cardio you did probably changed. Please detail the type and amount of cardio you were doing at the start (the first few weeks of your contest diet), halfway and final weeks of your contest prep.

**Initial Cardio**	**Middle Cardio**	**End Cardio**
Example: X minutes, low intensity cardio on a bike less than 60 % Max Heart Rate X times per week,	*Example: X minutes Y times per week, low intensity cardio on a bike less than 60 % Max Heart Rate,*	*Example: X minutes Y times per week, low intensity cardio on a bike less than 60% Max Heart Rate*
Example: X minutes Y times per week, low intensity cardio on a bike less than 60% Max Heart Rate,		

Resistance Training

Please detail the type and amount of resistance training you were doing at the start (the first few weeks of your contest diet), halfway and final weeks of your contest prep.

Initial Resistance Training	Middle Resistance Training	End Resistance Training
Example: 4 day split:<br>Chest and abs<br>Back<br>Shoulders and arms<br>Legs<br>1.5 hr per session, loads between 85 to 95% 1 RM, 5 exercises per bodypart,<br>3 to 8 reps per set	*Example: 5 day split:<br>Chest<br>Back<br>Shoulders<br>Legs<br>Arms<br>1 hr per session, loads between 75 to 85% 1 RM, 5 exercises per bodypart,<br>5 to 10 reps per set*	*Example: Power Hypertrophy 4 times per week<br>upper power<br>lower power<br>upper hypertrophy<br>lower hypertrophy<br>1 hr per session, loads between 75 to 85% 1 RM, 5 exercises per bodypart,<br>5 to 15 reps per set*

End of Questionnaire
