# Supplementary material for: Nutritional strategies of high level natural bodybuilders during competition preparation
Source: J Int Soc Sports Nutr. 2018 Jan 15;15:4. doi: 10.1186/s12970-018-0209-z (PMC5769537; doi:10.1186/s12970-018-0209-z)
Supplement: Additional file 2: — Dietary intake of competitors adjusted for bodyweight expressed as Medians and Interquartile Ranges. (PDF 815 kb) [file 12970_2018_209_MOESM2_ESM.pdf]

Additional File 2

Figure S1 A to D

Figure A

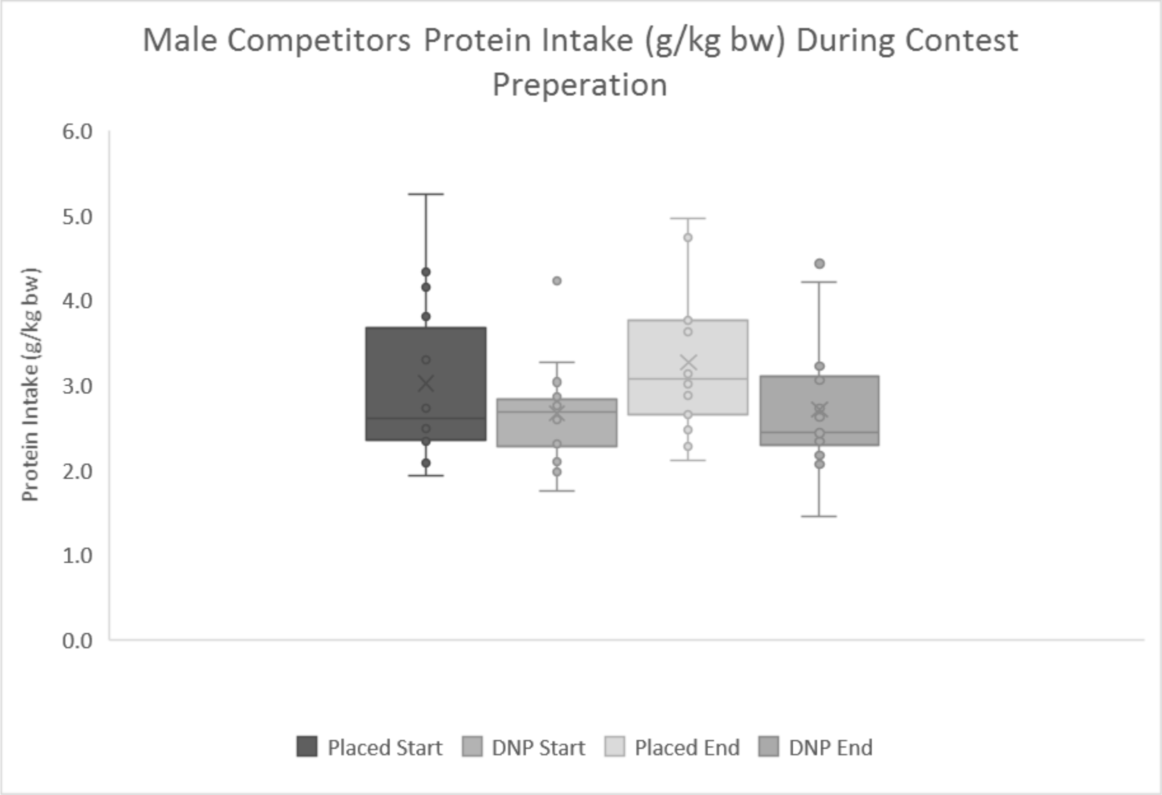

Figure B

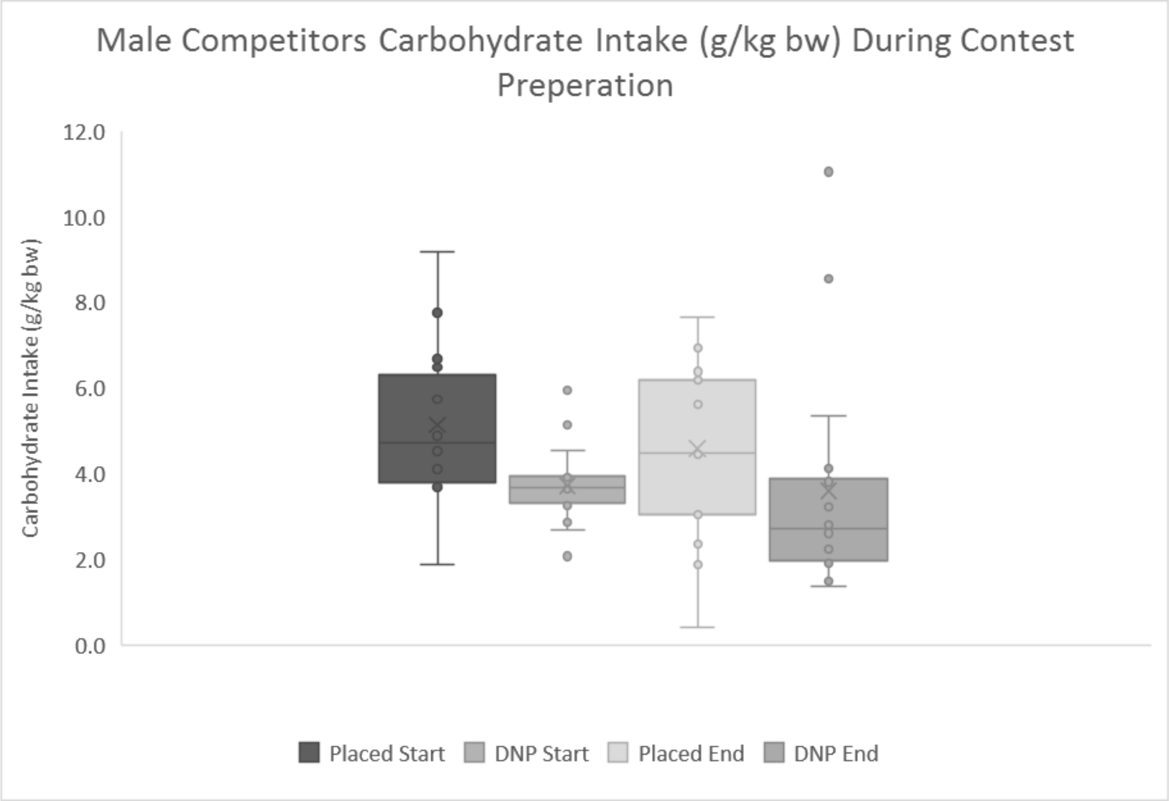

Figure C

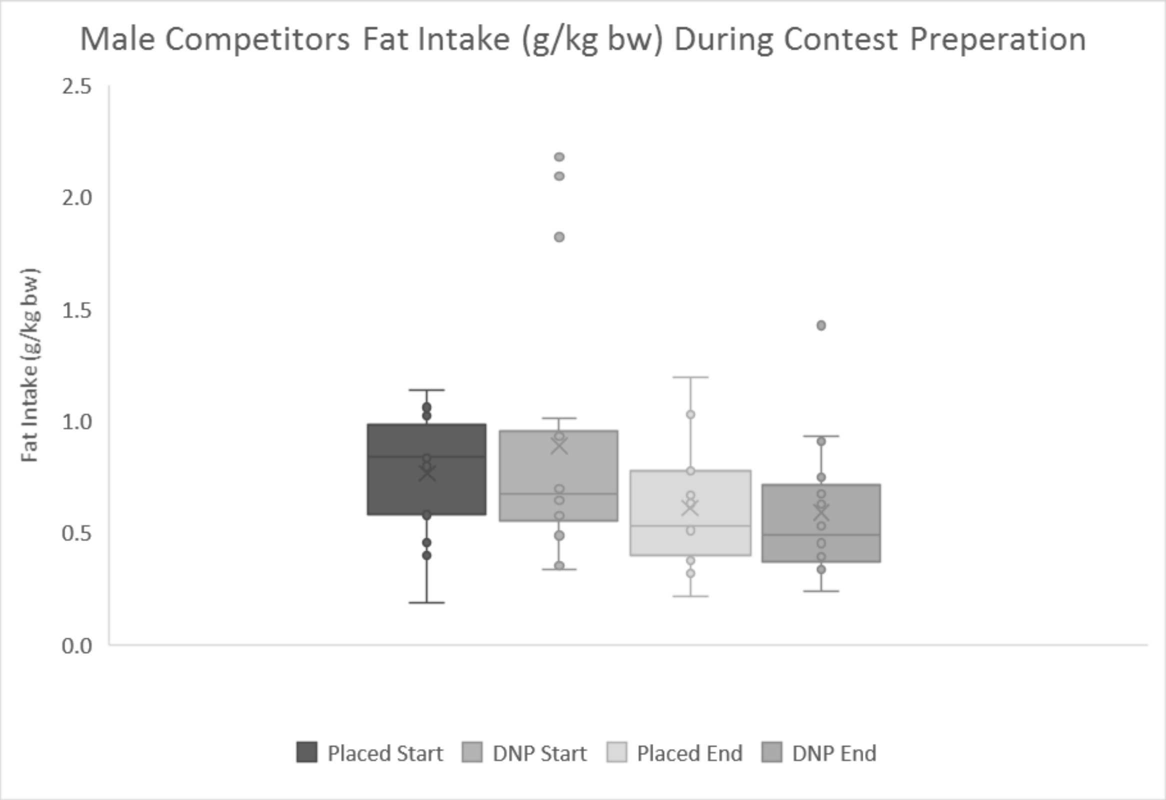

Figure D

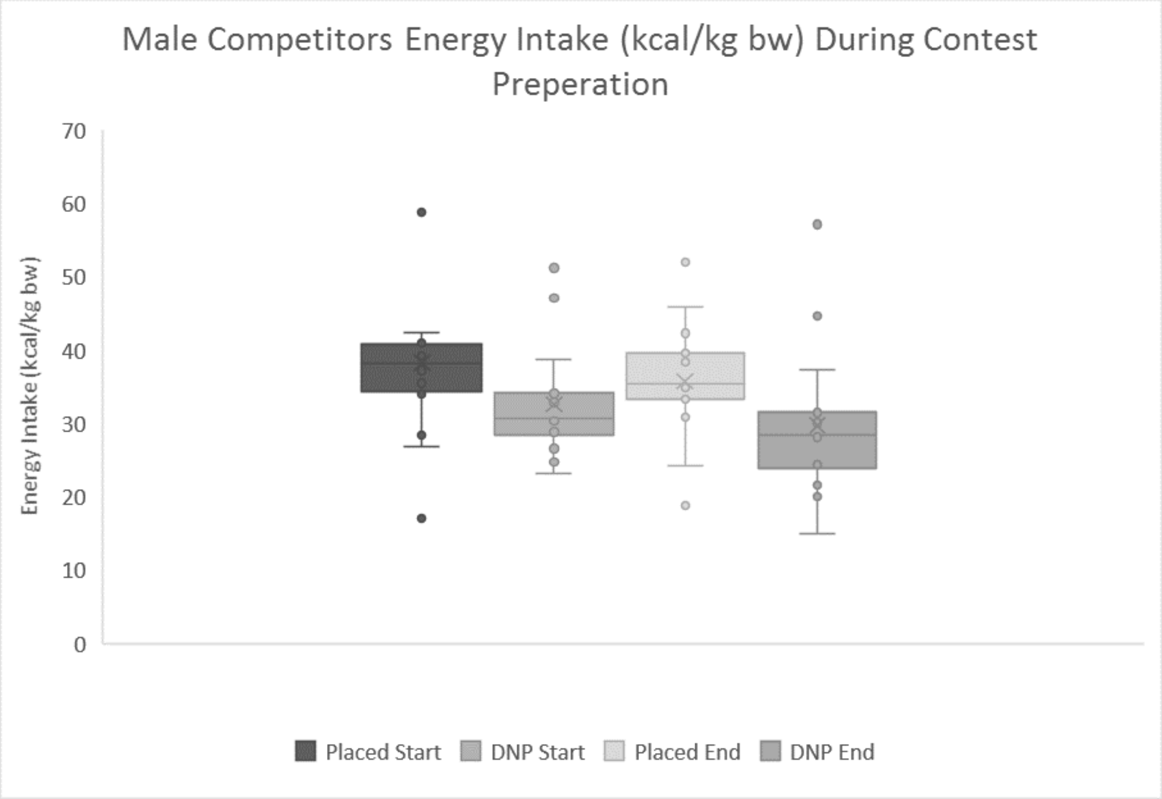

Figure S1 Macronutrient and Energy Intake Adjusted for Bodyweight of Male Bodybuilders during Contest Preparation: A *Protein intake g/kg BW* **B** *Carbohydrate intake g/kg BW* **C** *Fat intake g/kg BW* **D** *Energy intake kcal/kg BW. Box and whiskers indicate interquartile range. Symbols, × indicates mean, - indicates median, ○ individual data points, Abbreviations, Placed achieved top 5 of the competition, DNP did not place in top 5 of the competition. Start dietary intake at the start of competition diet, End dietary intake at the end of the competitive diet.*

Figure S2 A to D

Figure A

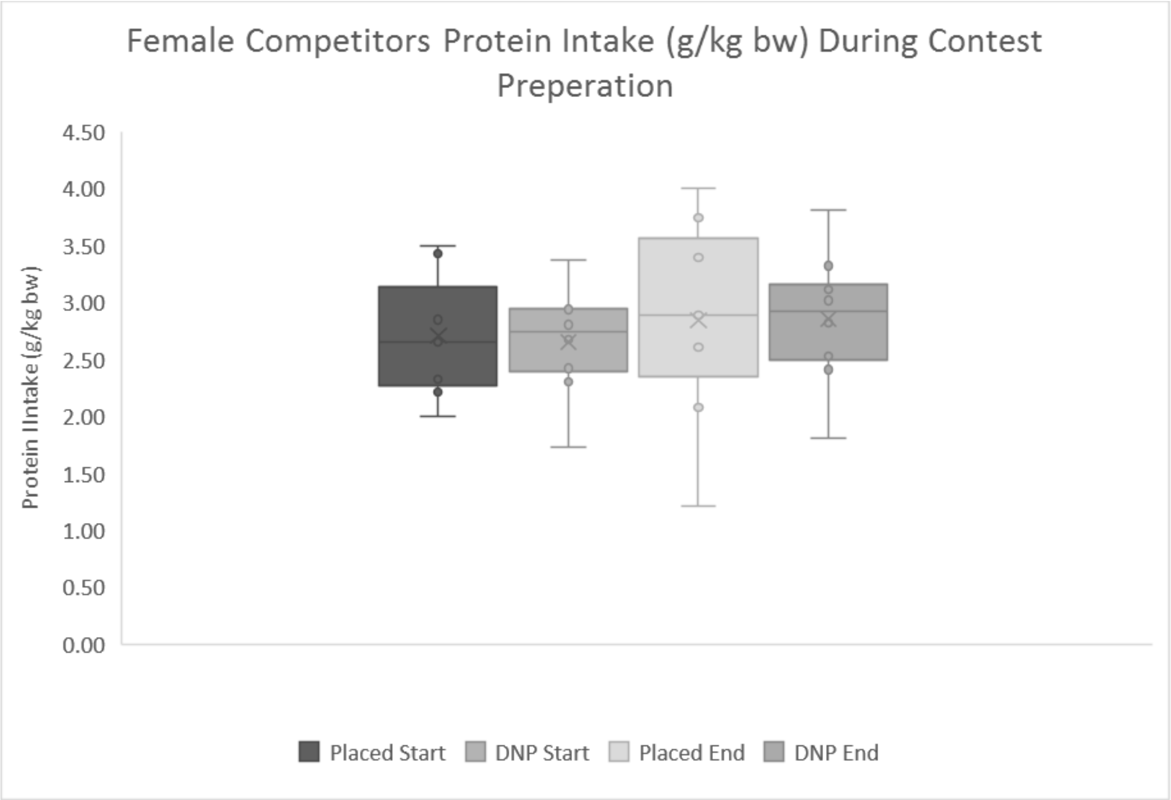

Figure B

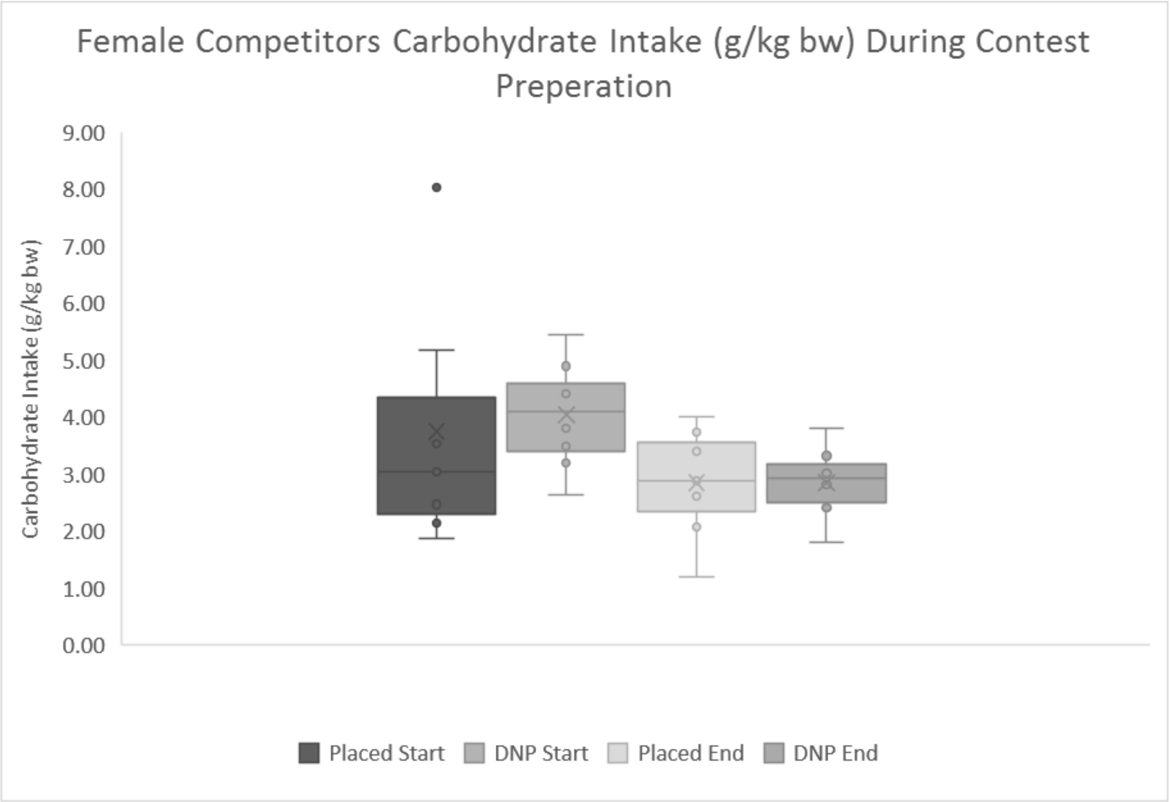

Figure C

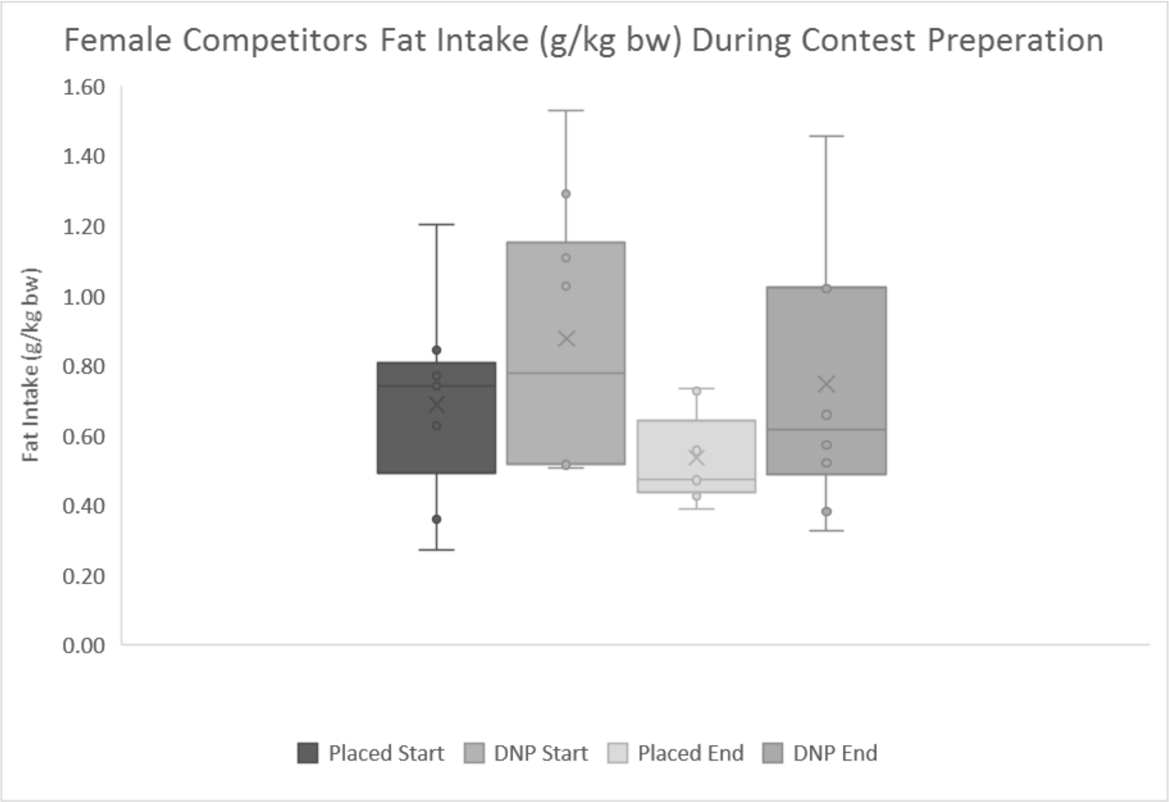

Figure D

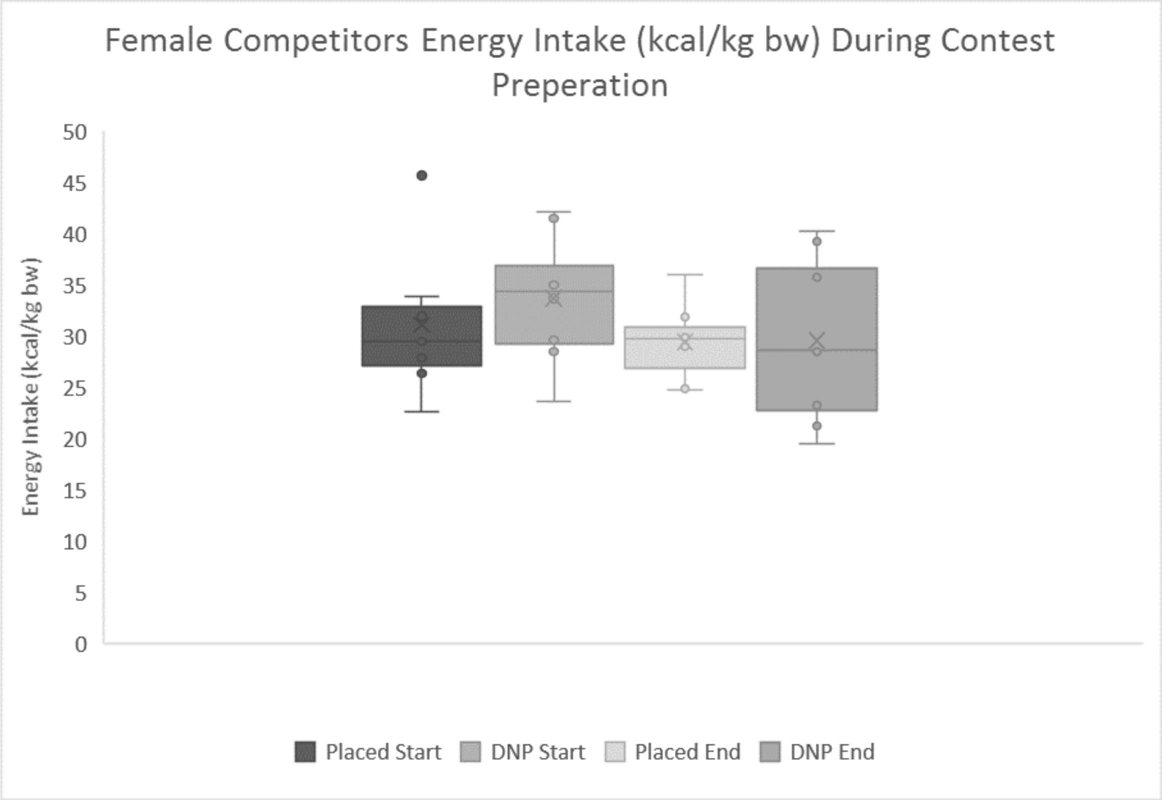

Figure S2 Macronutrient and Energy Intake Adjusted for Bodyweight of Female Bodybuilders during Contest Preparation: **A** *Protein intake g/kg BW* **B** *Carbohydrate intake g/kg BW* **C** *Fat intake g/kg BW* **D** *Energy intake kcal/kg BW. Box and whiskers indicate interquartile range. Symbols, × indicates mean, - indicates median, ○ individual data points, Abbreviations, Placed achieved top 5 of the competition, DNP did not place in top 5 of the competition. Start dietary intake at the start of competition diet, End dietary intake at the end of the competitive diet.*

Figure S3 A to D

Figure A

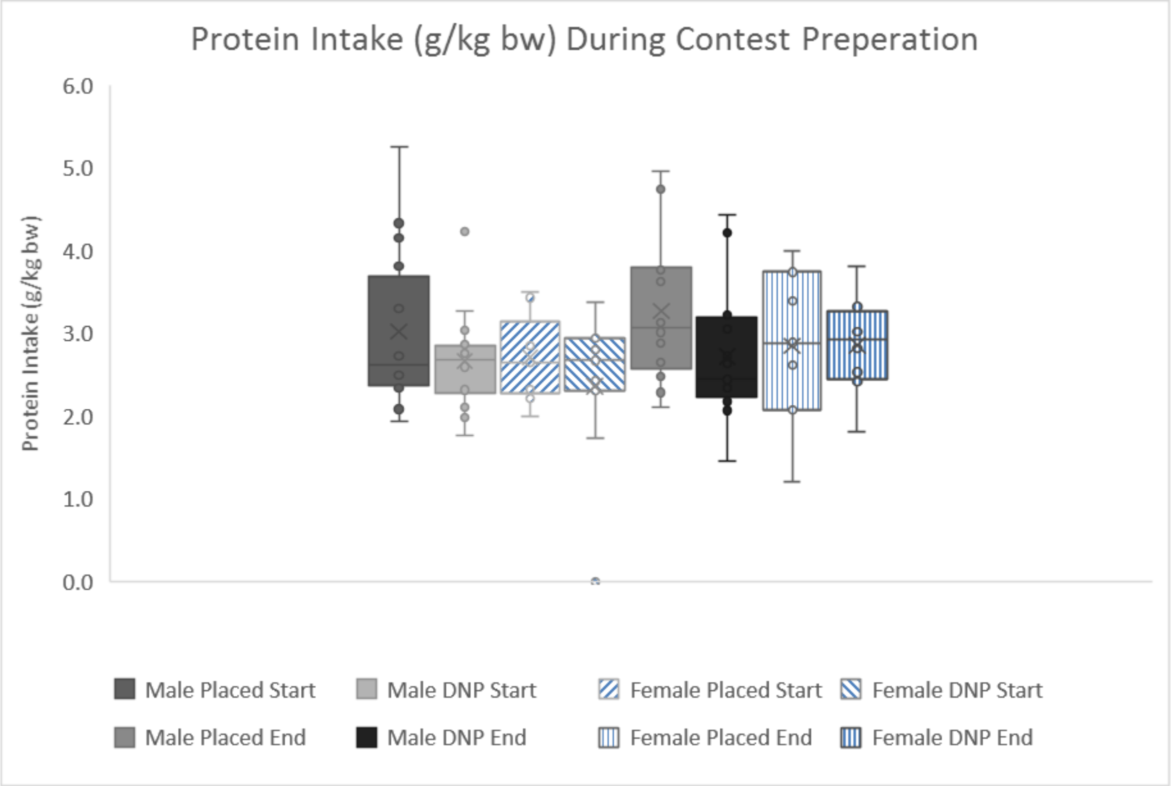

Figure B

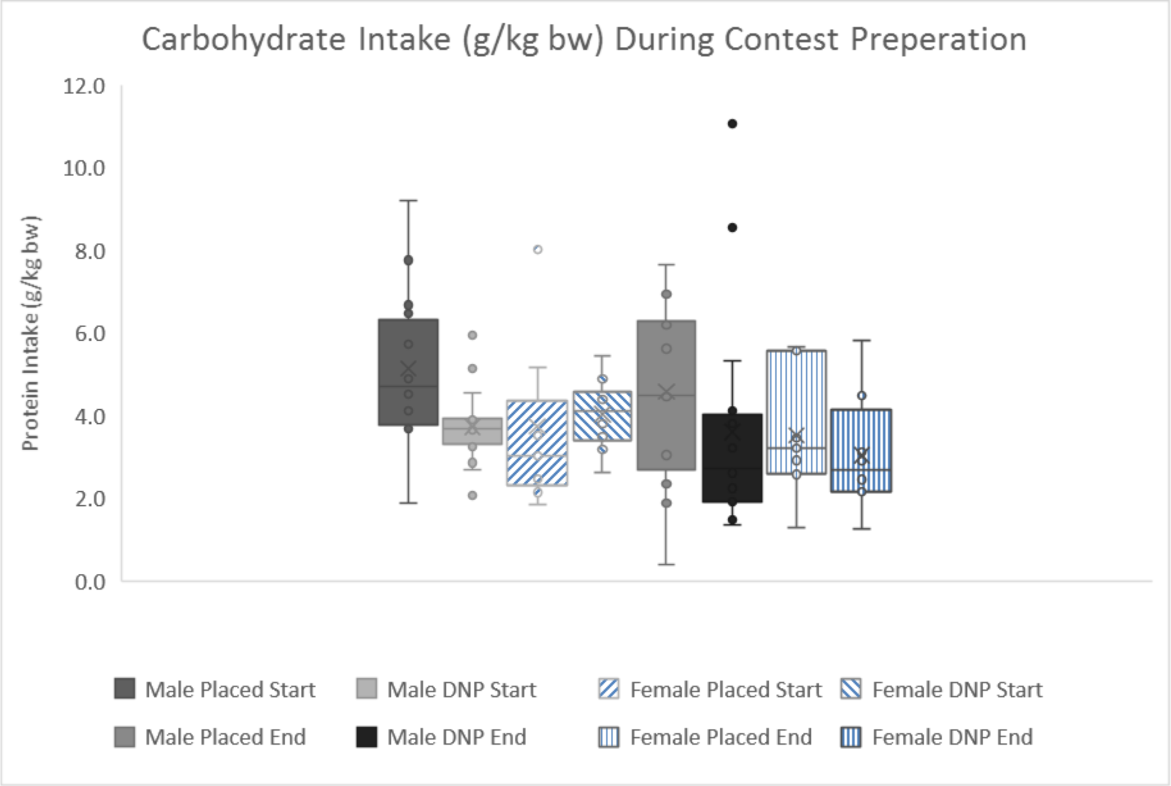

Figure C

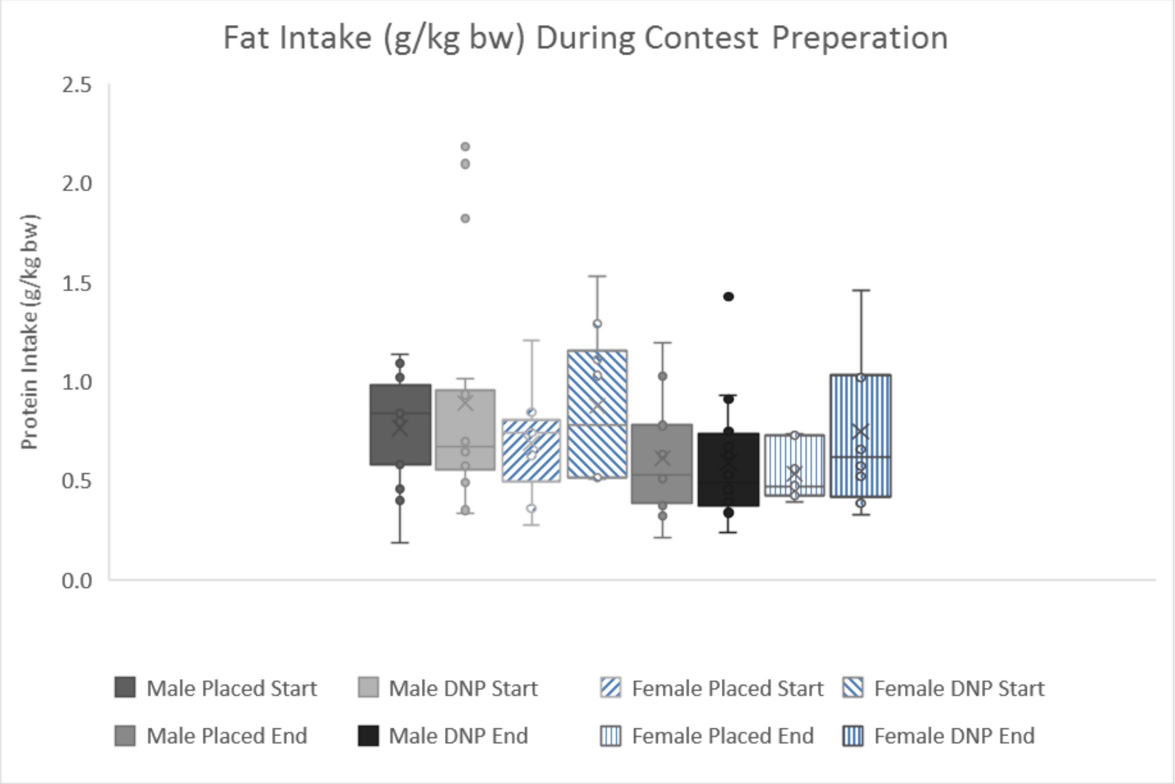

Figure D

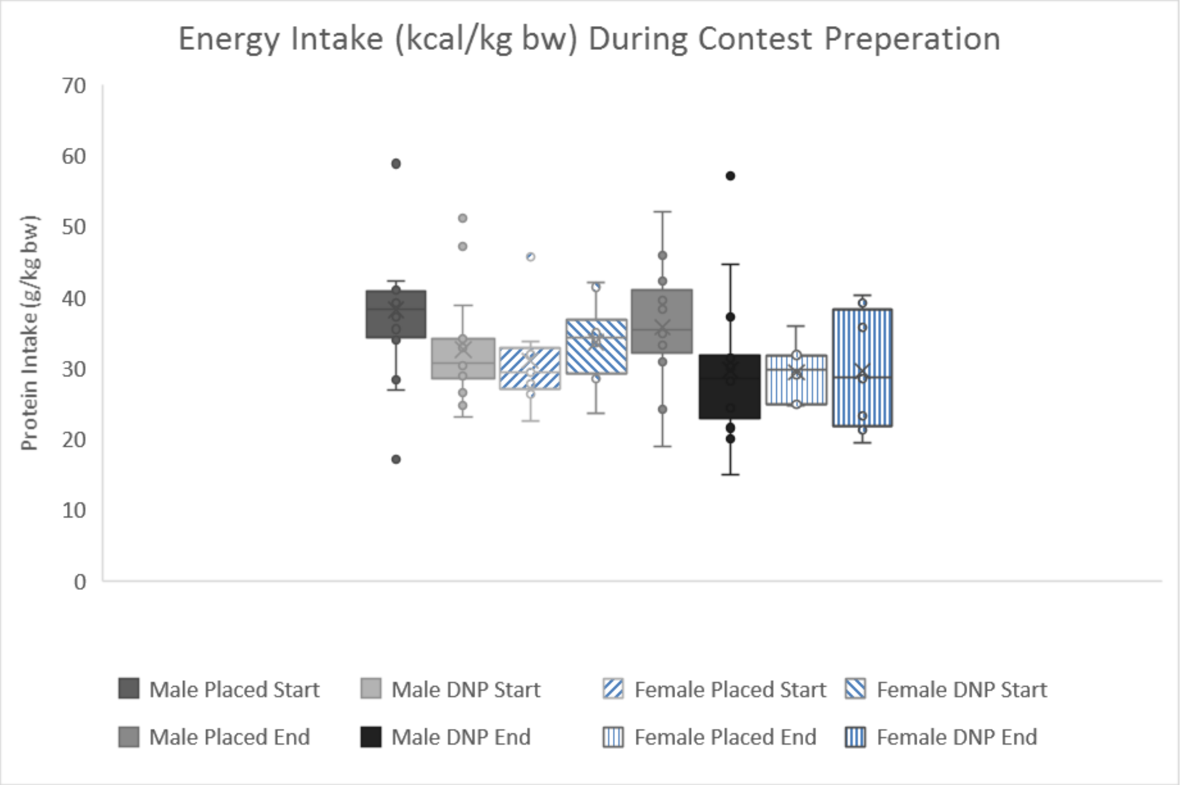

Figure S3 Macronutrient and Energy Intake Adjusted for Bodyweight of Male and Female Bodybuilders during Contest Preparation: A *Protein intake g/kg BW* **B** *Carbohydrate intake g/kg BW* **C** *Fat intake g/kg BW* **D** *Energy intake kcal/kg BW. Box and whiskers indicate interquartile range. Symbols, × indicates mean, - indicates median, ○ individual data points, Abbreviations, Placed achieved top 5 of the competition, DNP did not place in top 5 of the competition. Start dietary intake at the start of competition diet, End dietary intake at the end of the competitive diet.*
